# A combined strategies intervention on the World Health Organization prescribing indicators: A quasi-randomised trial

**DOI:** 10.4102/phcfm.v16i1.3943

**Published:** 2024-02-28

**Authors:** Nondumiso B.Q. Ncube, Tawanda Chivese, Ferdinand C. Mukumbang, Hazel A. Bradley, Helen Schneider, Richard Laing

**Affiliations:** 1 Department of Community and Health Sciences, School of Public Health, University of the Western Cape, Cape Town, South Africa; 2 Department of Population Medicine, College of Medicine, QU Health, Qatar University, Doha, Qatar; 3 South African Medical Research Council Health Services to Systems Research Unit, Cape Town, South Africa; 4 Boston University School of Public Health, Boston University, Boston, Massachusetts, United States of America

**Keywords:** rational drug prescribing, intervention, antibiotic resistance, Eswatini, rational medicine use, rational drug use

## Abstract

**Background:**

Irrational medicine use is a global problem that may potentiate antimicrobial resistance.

**Aim:**

This study aims to assess prescribing practices and the effect of a prescription audit and feedback coupled with small-group education intervention on prescribing indicators.

**Setting:**

The study was conducted in public-sector healthcare facilities in Eswatini.

**Methods:**

A cluster quasi-randomised controlled study was conducted from 2016 to 2019 using the World Health Organization/ International Network for Rational Use of Drugs (WHO/INRUD) prescribing indicators at baseline, post-intervention and post-follow-up. A 6-month unblinded intervention was tested in 32 healthcare facilities, randomly allocated to intervention (16) and control (16) arms. Prescribing practices were assessed post-intervention, and 6 months after the intervention, through an audit of 100 randomly selected prescriptions from each facility. Comparisons of WHO or INRUD prescribing indicators were conducted using the intention-to-treat analysis at the two times.

**Results:**

At baseline, in both arms, rational prescribing standards were met by the number of medicines per prescription and the use of injections. Antibiotic use was above 50% in both arms. After adjustment for baseline antibiotics use, region and level of care, there were no significant differences in all prescribing indicators between the two arms, post-intervention and at 6 months follow-up.

**Conclusion:**

In a lower middle-income setting with a high prevalence of irrational prescribing practices, a prescription audit, feedback and small-group education intervention had no benefits in improving rational prescribing.

**Contribution:**

Multifaceted strategies, strengthening of pharmacy and therapeutics committees, and holistic monitoring of medicine use are recommended to promote rational medicine use.

## Introduction

Irrational use of medicines is a global problem that results in the mismanagement of patients, and the wastage of critical resources, and has contributed to the growing burden of antibiotic resistance.^1,2^ As an effort to address irrational medicine use, indicators (core and complementary) for the evaluation of medicine use in health facilities were developed and validated by the World Health Organization (WHO).^3^ The core indicators are divided into prescribing, hospital, and patient care;^4,5^ and they have been reported to be less likely to change over time and context, more informative, and easier to perform.^6,7^

Various settings have used the WHO indicators to evaluate medicine use and prescribing practices. As there are no international standards that have been developed to assess prescribing, these WHO indicators are used as proxies.^6^ The core World Health Organization/International Network for Rational Use of Drugs (WHO/INRUD) prescribing indicators are average number of medicines (WHO standard = 2–3), average percentage of medicines prescribed by generic name (WHO standard = 100%), average percentage of prescriptions with one or more antibiotics (WHO standard ≤ 30%), average percentage of prescriptions with one or more injections (WHO standard ≤ 10%) and percentage of medicines prescribed from the essential medicines list (EML) (WHO standard = 100%).^2^

Although literature suggests that awareness on inappropriate prescribing is relatively high, medicines, particularly antibiotics, continue to be used irrationally. In low- and middle-income countries (LMICs), a 10-year review of empirical evidence showed that prescribing practices did not improve over time; the average number of medicines prescribed per patient increased from 2.1 to 2.8, and prescribing of antibiotics increased from 45% to 54%.^8^ A systematic review of prescribing practices conducted in 11 African countries showed that prescribing in the African region was not in line with WHO recommendations, more so in private than in public sector facilities.^6^ In Ethiopia, a study found that while the prescribing and dispensing practices were good, irrational use of antibiotics and the average number of medicines per prescription remained problematic.^9^ Irrational use of medicines has also been reported in other LMICs such as Sudan, Yemen, Thailand, Mali and Uganda.^10,11,12,13,14,15^

The success of interventions against irrational prescribing is debatable. Prescribing practices are influenced by educational, regulatory, economic and managerial factors.^2^ Managerial interventions are diverse and include limiting medicines available for prescribing in a setting, hence influencing the decisions that those prescribers would make,^16^ monitoring the use of guidelines and providing supervision, and auditing prescriptions and giving feedback to prescribers and pharmacists.^17^

There is evidence to support the use of multifaceted interventions in addressing irrational medicine use.^18^ A systematic review showed that a prescription audit and feedback intervention, a managerial strategy, improved healthcare professionals’ practice, although the effects were small to moderate.^19^ Other studies echo that interventions may have positive impacts on the rational use of medicines but those impacts tend to drop post-intervention.^20,21^

Minimal work on the rational use of medicines has been conducted in The Kingdom of Eswatini (previously Swaziland and hereafter referred to as Eswatini). In Eswatini, the WHO/INRUD indicators have not been integrated into the health system among the different levels of care, and hence, no prescribing targets and rates have been set for the five prescribing indicators. To provide initial data and the authors assessed prescribing indicators in the public sector and faith-based facilities across Eswatini. The authors then evaluated the effects of a short intervention, designed to improve WHO/INRUD prescribing indicators, immediately after the intervention and assessed whether the effects were sustained 6 months later. Findings from this study could be used to evaluate the applicability of the WHO/INRUD prescribing indicators in assessing prescribing in public sector facilities in Eswatini. Furthermore, findings could inform the need to validate the WHO/INRUD indicators for Eswatini and to allow for the development of different indicators to guide future prescribing in this setting, including prescribers’ compliance to the standard treatment guidelines.^22^

## Research methods and design

### Study design and sampling

A parallel cluster quasi-randomised controlled trial (Pan African Clinical Trial Registry [PACTR] registration number: PACTR202111807740758) was used to conduct this study, and the protocol followed is provided in Ncube 2020.^23^ The study was conducted in three phases: a baseline record review to assess prevailing prescribing patterns; intervention implementation and follow-up; and evaluation of prescribing practices at the end of the intervention to determine if the intervention had any effect on prescribing practices and whether intervention effects were sustained. The study was conducted between April 2016 and April 2019. A two-stage sampling strategy was employed to sample facilities (stage 1) and individual prescriptions (stage 2).

#### Sampling of facilities

A sample size calculation using 95% confidence interval, 5% *p*-value and 80% power gave us a sample size of 158. As this was a study towards a qualification with limited funding, it was not possible to conduct the study in all 158 facilities. Hence, the WHO/INRUD group’s recommendation of a minimum of 20 facilities to compare medicine use by facility was followed in conducting this study.^24^

The central medical store (CMS) provided a list of 325 public and faith-based facilities that received essential medicines from the CMS in 2016. The CMS codes facilities by region, and these codes were used to assign facilities to the four regions (Hhohho, Manzini, Lubombo and Shiselweni) in the country. Specialised facilities (i.e. national referral hospital, psychiatric hospital, tuberculosis [TB] hospital and those facilities governed by other bodies such as the police services and the army) were excluded from the sampling frame, leaving 286 eligible facilities. From a sampling frame of 286 facilities, the authors purposively selected one hospital per region (4) and all health centres in the country (5), leaving 277 clinics from which to sample to get the desired sample size. To sample clinics, a random sequence, generated by the principal investigator (N.B.Q.N) was used to select five clinics per region and inflated the sample of clinics per region by 20% (making six clinics per region) to account for non-response. The number of included facilities was 33; however, one facility (a clinic) in the Shiselweni region was unavailable for research activities because of reasons beyond the research team, making a total of 32 facilities.

#### Sampling of prescriptions

Prescriptions were included if they were complete with legible handwriting; if a prescription was illegible, the next legible prescription was included. Penicillins and other antibacterial agents, anti-infective ophthalmic and dermatologic agents and antidiarrheal agents (excluding agents reserved for use as anti-TB agents like streptomycin) were included. Prescriptions for antiretroviral therapy (ART), TB and family planning were excluded as there were parallel programmes capturing information on these. Outpatient prescriptions were sampled from pharmacies and records rooms in facilities, and data were collected according to what was prescribed not necessarily dispensed. To sample prescriptions in each facility, we followed Hogerzeil et al.’s^24^ recommendation of 100 outpatient encounters per facility to allow for comparisons between facilities. One hundred retrospective prescriptions for the periods, April 2016–March 2017 (baseline), October 2018 (immediate post-intervention) and March 2019 (end of the follow-up period), were randomly selected and extracted from the paper-based and electronic (client management information systems – CMIS) systems. At the end of the study, 25 of the 32 facilities (78%) had adopted the CMIS as opposed to only two facilities at baseline.

Further information on the sampling of prescriptions is available in a thesis submitted at the University of the Western Cape.^25^ A total of 3,200 outpatient prescriptions were randomly extracted from the 32 facilities at each of the three time periods.

### Setting

The study was conducted in randomly selected public sector and not-for-profit faith-based health facilities in Eswatini. Eswatini is a landlocked country in Southern Africa. The country is divided into four administrative regions: Hhohho, Manzini, Shiselweni and Lubombo. A three-tier health services delivery model operates in the country: (1) Clinics deliver primary healthcare services; prescribing and dispensing are handled by nurses. (2) Health centres deliver primary and secondary healthcare services; prescribing is handled by nurses and doctors, while pharmacy technicians manage and dispense medicines. (3) Hospitals deliver primary, secondary and specialised healthcare services; prescribing is handled by nurses, doctors and specialists, while pharmacists and pharmacy technicians manage and dispense medicines. A patient can choose to receive healthcare services at any service delivery level regardless of required services.

### Intervention design

Baseline results were used to inform the design of the intervention. The results showed that there was rampant usage of antibiotics across the country (Table 1). The intervention was designed around the problematic area identified during the baseline record review, that is, inappropriate use of antibiotics, where antibiotics had been used to manage non-communicable diseases (NCDs) such as diabetes, hypertension, asthma and arthritis without documentation of bacterial infection. Training material addressing the general use of antibiotics, with emphasis on no use of antibiotics for NCDs, was developed to be used during small-group onsite training sessions.

**TABLE 1:** Baseline World Health Organization/International Network for Rational Use of Drugs prescribing indicators in Eswatini.

Indicator	Intervention facilities†				Control facilities†				*P*
	*n*	%	mean	s.d.	*n*	%	mean	s.d.	
**Level of care**									
Level of care, primary	12	75.0	-	-	12	75.0	-	-	-
Level of care, secondary	4	25.0	-	-	4	25.0	-	-	-
**Region**									
Hhohho	6	37.5	-	-	4	25.0	-	-	-
Manzini	5	31.3	-	-	3	18.8	-	-	-
Shiselweni	1	6.3	-	-	7	43.8	-	-	-
Lubombo	4	25.0	-	-	2	12.5	-	-	-
**WHO/INRUD prescribing indicators**									
Average number of medicines	-	-	3.89	1.04	-	-	3.5	0.57	0.194
%Medicines prescribed by generic name	-	-	72.88	10.98	-	-	74.81	7.53	0.565
%Prescriptions with antibiotics	-	-	53.63	12.81	-	-	55.06	10.19	0.728
%Prescriptions with injections	-	-	10.69	9.51	-	-	8.69	5.21	0.466
%Medicines prescribed from the EML	-	-	94.06	3.19	-	-	95.06	2.71	0.348

† , *N* = 16 facilities, 1,600 prescriptions.

EML, essential medicines list; s.d., standard deviation.

### Allocation of facilities to intervention and control arms

Baseline survey results on antibiotics use were used to rank facilities from best to worst performing. The WHO/INRUD recommended standard on the use of antibiotics is that it should be ≤ 30%.^4^ Best-performing facilities were those that had the lowest percentage of use of antibiotics, while the worst-performing facilities were those that used more antibiotics. After ranking the facilities, random numbers generated in Excel by NBQN were used to allocate facilities to the intervention and control arms. On the list of random numbers, for each pair of intervention and control facilities, the higher percentage of use of antibiotics was allocated to an intervention and the lower number to the control facility. This was done to pair similar performing facilities (16 facilities in the intervention arm and 16 in the control arm).

The intervention was piloted in March 2018 in facilities that were not part of the study to allow researchers to assess the feasibility of delivering the intervention and identify processes that needed to be clarified and simplified. Piloting also allowed the research team to adequately plan for the effective implementation of the intervention in the included facilities.

### Intervention roll-out

Two visits, 6 months apart, to each intervention facility were conducted between May and September 2018. A similar supervision study conducted in Zimbabwe^21^ showed that two visits were enough to make a positive impact. At the end of the intervention, a 6-month follow-up period (October 2018 – March 2019), during which there were no visits to neither intervention nor control facilities, was implemented. During the first intervention visit, onsite collection and analyses of 30 randomly selected prescriptions from the previous month were performed. Results from the onsite analyses were presented to a small group of frontline facility managers (prescribers – doctors and nurses, pharmacists and pharmacy technicians) and compared with baseline facility results. Intensive discussions around optimal and problematic prescribing were held with the small group. Supportive supervision on appropriate prescribing, including education on the management of asthma, diabetes, hypertension and arthritis, was given. Similar activities were performed during the second visit.

At the end of the follow-up period, evaluation data for two time points, namely, immediate post-intervention (October 2018) and post-follow-up (April 2019), were collected.

### Statistical analyses

The WHO/INRUD prescribing indicators were tested for normality at each time point using the Shapiro–Wilks test. Medians and interquartile ranges (IQR) were reported for WHO/INRUD prescribing indicators, and the Wilcoxon rank sum test was used to compare the WHO/INRUD prescribing indicators at the three time points. The WHO/INRUD prescribing indicators that were not normally distributed were the percentages of injections and medicines prescribed from the EML (at baseline), percentages of prescriptions with injections and generic prescribing (post-intervention) and all indicators but percentage of prescriptions with antibiotics at the end of the follow-up period. After linear regression, residuals were tested for normality using the Shapiro–Wilks test. Residuals for the prescribing indicators were normally distributed except for the percentage of prescriptions with injections at the follow-up. Model specification was tested using the link test. For each regression model, we adjusted for the baseline percentage of prescriptions with antibiotics, region and level of care. The full regression models and diagnostics are in Online Appendix 2. An alternative multivariate linear regression was conducted and presented as Online Appendix 1, Table 1 and Table 2. Facilities were analysed according to the group they were allocated to using the intention to treat principle. All analyses were conducted in Stata (version 15).

**TABLE 2:** Comparison of World Health Organization/International Network for Rational Use of Drugs prescribing indicators at post-intervention and post-follow-up by intervention and control facilities in Eswatin (intention to treat analyses).

Factor	Control (*N* = 16)		Intervention (*N* = 16)		*P*
	mean	IQR	mean	IQR	
**Post-intervention**					
Average no of medicines	3.3	2.8, 3.8	3.5	3.2, 3.9	0.46
% Medicines prescribed by generic name	*87.5*	*80.5, 90.0*	*80.5*	*72.0, 86.5*	*0.04*
% Prescriptions with antibiotics	44.5	32.5, 61.5	53.5	42.5, 60.0	0.29
% Prescriptions with injections	6.0	3.5, 9.5	6.5	4.0, 13.5	0.75
% Medicines prescribed from the EML	*92.0*	*90.0, 95.0*	*90.5*	*85.0, 92.0*	*0.04*
**Post-follow-up**					
Average no of medicines	3.3	3.0, 3.8	3.5	3.0, 3.8	0.62
% Medicines prescribed by generic name	88.0	84.5, 91.5	85.5	76.5, 87.5	0.06
% Prescriptions with antibiotics	57.0	50.0, 66.0	52.5	48.5, 63.0	0.52
% Prescriptions with injections	7.0	3.5, 18.0	5.5	2.5, 15.5	0.56
% Medicines prescribed from the EML	94.0	91.0, 96.5	93.0	87.0, 94.0	0.12

Note: Values in italics are statistically significant results.

IQR, interquartile ranges; EML, essential medicines list.

### Ethical considerations

Ethics clearance was granted by the University of the Western Cape Higher Degrees Committee (reference BM/16/4/2) and the National Health Research Review Board in Eswatini. Permission to access healthcare facilities was granted by the office of the Deputy Director of Pharmaceutical Services in the Ministry of Health in Eswatini. The study posed a minimal risk as we collected data from records and removed all identifiers during analyses. The intervention did not pose risks for healthcare practitioners. Consent was not sought from participants as data were collected from patient records. However, permission to access facility records was granted by the office of the Deputy Director of Pharmaceutical Services and the Regional Health Administrators in the four regions of Eswatini.

### Role of the funding source

The study sponsors had no role in the conduct of the study. The principal investigator –NBQN, Tawanda Chivese (TC), Hazel Bradley (HB), Richard Laing (RL) and Helen Schneider (HS) had access to the data. The principal investigator took the decision to submit the manuscript for publication.

## Results

**FIGURE 1:**
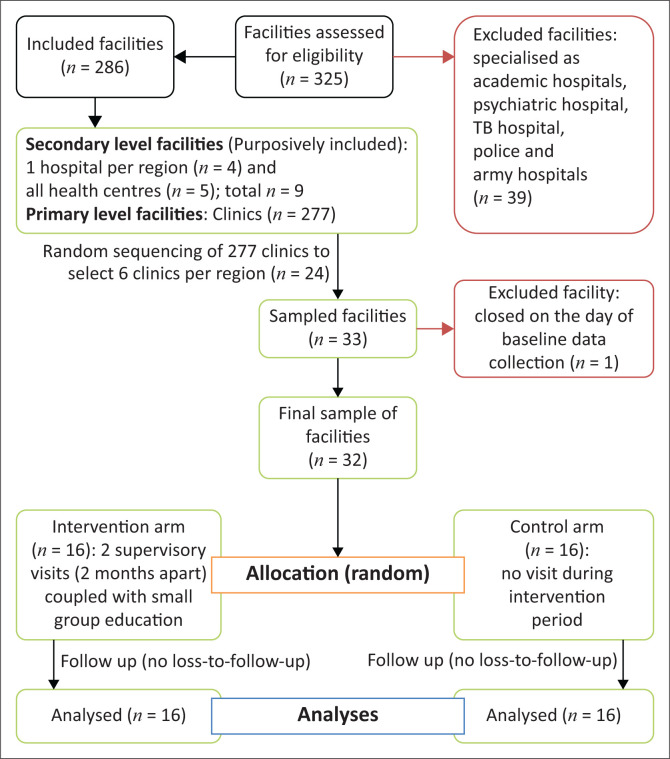
Consort diagram showing sampling, allocation and analyses of facilities.

Figure 1 shows the sampling of facilities, their allocation to intervention and control arms and the total number of facilities analysed at the end of the study.

A total of 3,200 prescriptions were analysed at baseline. The intervention arm had 16 facilities: 6 (38%) in the Hhohho region, 1 (6%) in Shiselweni, 3 (19%) in Lubombo and 6 (38%) in the Manzini region. The control arm also had 16 facilities: 4 (25%) in the Hhohho region, 7 (44%) in Shiselweni, 2 (13%) in Lubombo and 3 (19%) in the Manzini region as shown in Table 1. Table 1 also reports baseline findings of the WHO/INRUD prescribing indicators by intervention and control facilities. Rational prescribing standards as recommended by WHO were only met by the average number of medicines per prescription (though the average was slightly higher than the standard 2–3 medicines per prescription) and the percentage of prescriptions with injections of 10.7% (though slightly higher than the WHO standard of ≤ 10%), in both intervention and control facilities (Table 1). The other prescribing indicators did not reach the recommended standards in both intervention and control facilities (Table 1). Of note at baseline was the high use of antibiotics, above 50%, in both intervention and control facilities (the WHO recommendation is for the average percentage of prescriptions with one or more antibiotics to be ≤ 30%) (Table 1).

A total of 3,200 post-intervention and 3,200 post-follow-up prescriptions were analysed at the end of the study. There were no deviations from the intervention protocol, that is, facilities were analysed in the arms to which they were allocated.

### Comparisons of World Health Organization/International Network for Rational Use of Drugs prescribing indicators post-intervention and at the end of the follow-up period by intervention and control facilities

Immediately after the intervention, the percentage of medicines prescribed by generic name (median = 80.5; IQR = 72.0 – 86.5; *p* = 0.04) and the percentage of medicines prescribed from the EML (median = 90.5; IQR = 85.0 – 92.0; *p* = 0.04) were statistically significantly higher in the intervention facilities (Table 2). There were no statistically significant differences between the intervention and control facilities in the average number of medicines per prescription and the percentages of prescriptions with antibiotics and injections. At the end of the follow-up period, there were no statistically significant differences in all the WHO/INRUD prescribing indicators between the intervention and control facilities (Table 2).

### Effect of the intervention immediately after the intervention and at 6 months after the intervention – Multiple variable linear regression

For the time point immediately after the intervention, after adjusting for the baseline percentage of prescriptions with antibiotics, region and level of care, the percentage of medicines prescribed from the EML (coefficient = −3·13; 95% CI = −5·98 – −0.27; *p* = 0·03) were statistically significantly lower in the intervention compared to the control facilities. At the same time point, there was no significant effect of the intervention on all the other prescribing indicators (Table 3). At the end of the follow-up period, there was also no statistically significant effect of the intervention on the five prescribing indicators (Table 3).

**TABLE 3:** Effect of the intervention immediately after the intervention and at 6 months after the intervention – multiple variable linear regression.

Prescribing indicator	Coefficient	*P*	95% confidence interval
**Post-intervention (immediately after the intervention)**			
Average number of medicines	0.11	0.67	−0.43 to 0.65
% Medicines prescribed by generic name	−5.17	0.15	−12.38 to 2.04
% Prescriptions with antibiotics	5.16	0.38	−6.76 to 17.09
% Prescriptions with injections	1.01	0.64	−3.41 to 5.43
% Medicines prescribed from EML	*−3.13*	*0.03*	*−5.98 to −0.27*
**Follow-up (6 months after the end of intervention)**			
Average number of medicines	0.03	0.90	−0.51 to 0.58
% Medicines prescribed by generic name	−3.61	0.30	−10.57 to 3.35
% Prescriptions with antibiotics	−1.99	0.64	−10.75 to 6.76
Log % prescriptions with injections	−0.16	0.69	−1.00 to 0.68
% Medicines prescribed from EML	−2.46	0.15	−5.89 to 0.96

Note: Values in italics are statistically significant results.

Each model is adjusted for the baseline percentage of prescriptions with antibiotics, region and level of care. Analysis is according to the intention to treat. The percentage of prescriptions with injections at follow-up were transformed using the log transformation.

EML, essential medicines list.

## Discussion

At baseline, we found a high prevalence of irrational prescribing practices, particularly overuse of antibiotics. The percentage of prescriptions with one or more antibiotics prescribed were higher than 50%, in both intervention and control facilities. A prescription audit, feedback and small-group education intervention had no effect on the five WHO/INRUD prescribing indicator standards.

Findings showed that WHO/INRUD prescribing indicators were outside the recommended WHO standards immediately after the intervention and 6 months after the intervention. The high use of antibiotics in this LMIC is of concern as this could promote antimicrobial resistance (AMR). This high use of antibiotics in Eswatini could be because of the high burden of infectious diseases such as diarrhoea and sexually transmitted diseases. In this setting, the availability of microbiology testing services is poor, and this may result in the prescribing of antibiotics even for conditions that do not require them. Interventions targeted at increasing microbiology testing and human resources for this testing need to be prioritised to minimise the management of non-bacterial conditions with antibiotics.

With growing global evidence on the spread of multidrug-resistant bacteria, the country could soon run out of treatment options for bacterial infections. O’Neill^26^ states that if proactive solutions to curb the spread of AMR are not provided, an estimated 10 million lives a year globally are at risk of suffering drug-resistant infections.^1,26^ Apart from irrational antibiotic prescribing, the high number of medicines per prescription shows a high prevalence of polypharmacy in the country, which may be because of increasing co-morbidities in Eswatini. This study’s findings showed irrational prescribing as generic prescribing, prescribing of medicines from the EML and prescribing of injections were also out of recommended WHO standards. Therefore, there is a need for Eswatini to strengthen policies and interventions that promote rational use of medicines.

A small reduction, though not statistically significant, in the use of antibiotics in the intervention arm was observed at the end of the follow-up period, showing that, though not evident immediately post-intervention, the intervention might be beneficial in reducing the use of antibiotics. More research over a longer period needs to investigate whether this can be sustained in Eswatini. Regional pharmacists who were appointed in 2018,^27^ after the presentation of baseline study findings to senior officials under pharmaceutical services, could incorporate the prescription audit and feedback sessions into their supervisory and mentorship visits. There is a possibility that if the intervention is sustained over a longer time, there would be an effect in improving rational prescribing. The effect of system and policy changes could be evaluated using interrupted time series. Furthermore, other interventions that have been shown to reduce antibiotic use in literature still need to be explored in Eswatini. These include the availability of policies that will advocate for the implementation of a national AMR containment strategy, availability of an entity that will provide information on medicines to healthcare professionals and the community, and availability of a functional department dedicated to promoting rational use of medicines within the Ministry of Health which will ensure functional pharmacy and therapeutics committees (PTCs) and antimicrobial stewardship (AMS) committees in health centres, hospitals and regions.^28^ A good intervention to promote rational use of antibiotics is the establishment and use of AMS committees that can be constituted at all levels of care.^29,30^

Antimicrobial stewardship committees improve the use of antibiotics through facilitating timely microbiology and antimicrobial susceptibility testing (AST) and proper patient management.^31^ Antimicrobial stewardship committees could also establish surveillance mechanisms to monitor antibiotics use to generate information that can be used to produce facility antibiograms that can influence antibiotics use in specific facilities; in turn, national AMR data could be used to influence policies and treatment guidelines.^32^ Studies to further assess antibiotics that Eswatini uses according to the WHO access, watch reserve (AWaRe) classification are needed.

Although the study combined an educational and managerial intervention to influence prescribing in line with recommendations from literature to use multifaceted interventions, the intervention had no effect on prescribing practices. This has also been noted in other settings, where irrational prescribing of antibiotics did not go down significantly after intervention.^33,34,35^ In this study, this could have been because of several factors. Prescribing behaviour is a complex behaviour that is influenced by both intrinsic factors such as education, attitudes, experience, complacency and sociodemographic factors and extrinsic factors such as patient-related factors, health system and costs.^36^ A qualitative study conducted in Eswatini showed that system (stockouts of essential medicines), patient (pressure from patients to be prescribed many medicines) and provider (prescribers prescribing as many medicines as possible to reduce visits to the facility and reduce patient volumes) factors impact prescribing practices.^25^ Therefore, it may require more time and more intensive interventions to change prescribing behaviours. Further, the feedback meeting held with all facilities at the end of the baseline survey provided both control and intervention personnel with educational aspects of the intervention and may have influenced behaviour in both arms, as can be seen in the reduction in the percentages of prescriptions with antibiotics and injections in both intervention and control facilities at post-intervention and post-follow-up time points. Lastly, prescribers were aware of future assessments across both arms and could have changed their prescribing behaviour in anticipation.

Although we observed no effect of the intervention in this study, this study has shown that a small-group education intervention coupled with prescription audit and feedback is feasible in a LMIC. Future studies need to assess the use of other interventions. A limitation of the WHO/INRUD prescribing indicators is that they may not be sensitive in detecting changes in particular LMIC settings, as was shown in a previous study in Namibia.^37^ The Namibian study tested the sensitivity of each of the WHO/INRUD indicators and found that sensitivity rates of these indicators ranged from 11.6% to 72.3%.^37^ Other studies may be required to assess the applicability of the WHO/INRUD prescribing standards in Eswatini. Also, additional indicators, such as prescribers’ compliance to the standard treatment guidelines (STGs), to guide and assess the quality of future prescribing based on the WHO definition of rational medicine use are needed in the country. There may be a need for Eswatini to develop thresholds for the WHO/INRUD prescribing indicators so that future studies may compare against these. This study may provide initial data thresholds for these indicators. If the assumption would be that after the intervention prescribers were prescribing rationally, data from this study could be used as a basis for the development of thresholds that regional pharmacists can use during their mentorship and supportive supervision visits. However, more studies are required.

Another limitation of our study is that we did not assess the long-term durability of the intervention.

Lastly, staff rotations may have affected the effect of the intervention, and the introduction of an electronic system, CMIS, may have affected the performance of the intervention. The electronic system is preloaded with medicines both in trade and generic names. Also, prescribers can choose any treatment for any diagnosis, that is, after selecting the diagnosis, CMIS does not limit the medicines that one can select to manage that condition. This allows prescribers to select antibiotics to manage NCDs. Assessments on whether the information on CMIS is aligned to information in the Standard Treatment Guidelines and EML of Common Medical Conditions in the Kingdom of Eswatini need to be conducted.

## Conclusion

In a lower-middle-income setting context with a high prevalence of irrational prescribing practices, a prescription audit, feedback and small-group education intervention did not have benefits in improving rational antibiotic prescribing. System changes such as the introduction of an electronic prescribing and dispensing system, rotation of staff members between intervention and control facilities and the appointment of regional pharmacists during the study could have influenced the performance of the intervention. The implementation of the intervention by the researcher and not as a service integrated into facility processes could have also affected the performance of the intervention.^25^

### Contribution

The authors recommend multifaceted strategies including education of communities, frontline medicine managers (physicians, doctors, nurses and pharmacy personnel), supervisors and policymakers on the rational use of medicines; strengthening of facility PTC and the role and availability of pharmacy personnel in support of rational medicine use at all levels of care; and developing an ongoing system of data collection and analyses as well as monitoring and evaluation (M&E) of medicine use at facility, regional and national levels to support targeted interventions for promoting the rational use of medicines.^4,38,39^
